# Acute Effects of Mental Recovery Strategies After a Mentally Fatiguing Task

**DOI:** 10.3389/fpsyg.2020.558856

**Published:** 2020-12-23

**Authors:** Fabian Loch, Annika Hof zum Berge, Alexander Ferrauti, Tim Meyer, Mark Pfeiffer, Michael Kellmann

**Affiliations:** ^1^Unit of Sport Psychology, Faculty of Sport Science, Ruhr University Bochum, Bochum, Germany; ^2^Unit of Training and Exercise Science, Faculty of Sport Science, Ruhr University Bochum, Bochum, Germany; ^3^Institute of Sports and Preventive Medicine, Saarland University, Saarbrücken, Germany; ^4^Institute of Sport Science, Johannes Gutenberg University Mainz, Mainz, Germany; ^5^School of Human Movement and Nutrition Sciences, The University of Queensland, St Lucia, QLD, Australia

**Keywords:** fatigue, psychological, performance, rest, cognitive, mental break

## Abstract

Both daily demands as well as training and competition characteristics in sports can result in a psychobiological state of mental fatigue leading to feelings of tiredness, lack of energy, an increased perception of effort, and performance decrements. Moreover, optimal performance will only be achievable if the balance between recovery and stress states is re-established. Consequently, recovery strategies are needed aiming at mental aspects of recovery. The aim of the study was to examine acute effects of potential mental recovery strategies (MR) on subjective-psychological and on cognitive performance outcomes after a mentally fatiguing task. A laboratory-based randomized cross-over study with twenty-four students (22.8 ± 3.6 years) was applied. Participants were run through a powernap intervention (PN), a systematic breathing intervention (SB), a systematic breathing plus mental imagery intervention (SB+), and a control condition (CC) with one trial a week over four consecutive weeks. Mental fatigue was induced by completion of the 60-min version of the AX-continuous performance test (AX-CPT). The Short Recovery and Stress Scale (SRSS) and Visual Analog Scales (VAS) were assessed to measure effects on perceptual outcomes. Cognitive performance was measured with a reaction time test of the Vienna Test System (VTS). During all three recovery interventions and CC portable polysomnography was applied. Results showed a significant increase from pre-AX-CPT to pre-MR on fatigue states and recovery-stress states indicating that the induction of mental fatigue was effective. Moreover, results underlined that analysis yielded no significant differences between recovery interventions and the control condition but they revealed significant time effects for VAS, SRSS items, and cognitive performance. However, it could be derived that the application of a rest break with 20 min of mental recovery strategies appears to enhance recovery on a mainly mental and emotional level and to reduce perceived mental fatigue.

## Introduction

It is well-established that the impact of a high workload as well as extensive cognitive and emotional demands over a prolonged period of time can lead to mental fatigue and increased emotional exhaustion (Sonnentag and Fritz, 2015). Moreover, the emerging feeling of “*I need a short rest break”* is known by everyone in their daily life. Relating to this, rest breaks, as a countermeasure, can be applied to recover on a regular basis from daily demands and enable to replenish mental and physical resources (De Bloom et al., 2017). Research on effects of rest breaks in occupational psychology illustrates that rest breaks can curb the increase of fatigue (i.e., mental and physical fatigue) associated with high work demands with the aim to maintain the necessary levels of focus and engagement over time (Sonnentag and Fritz, 2007; Bakker, 2011). In addition, rest breaks have been found to reduce subjective perception of fatigue and improve performance in demanding situations (Blasche et al., 2018). Research in the field of occupational health has demonstrated that strategies like breathing techniques, mental imagery, and powernaps appear to have positive effects on mental states such as concentration, attention, vigilance as well as on performance (Sonnentag and Fritz, 2007; De Bloom et al., 2017).

Transferring such insights to the sporting context, especially multisport events such as shooting, swimming, modern pentathlon, or track cycling consisting of multiple intensive competition bouts in a single day (e.g., qualification heat and final, distinct contests) can result in specific physical (e.g., specific physiological and technical requirements) and mental demands (e.g., sustained concentration, attentional control, perceptual skills; Le Meur et al., 2010; Nédélec et al., 2012; Ortega and Wang, 2018). These specific characteristics of all-day competitions appear to be mentally demanding and can lead to an acute state of mental fatigue (Coutts, 2016; Kellmann et al., 2018).

Regarding this, two largely independent lines of research with the focus on mental fatigue as well as on ego depletion have developed in the research fields of psychology and exercise physiology, which address the same body of research, although using different explanations (Giboin and Wolff, 2019).

The state of ego depletion is based on the strength model of self-control that explains performance decrements due to the prior exertion of mental effort or self-control demands (e.g., attention, regulation; Englert, 2017). All acts of self-control are based on a single energy resource (i.e., self-control strength), that is assumed to have a limited capacity and can become temporarily depleted. This state of depleted self-control resources is called ego depletion which may lead to the fact that athletes are less persistent during strenuous physical exercise or tend to perform worse under pressure (Englert, 2016, 2017). In comparison to this, however, the focus of the present paper will be pre-dominant on the model of mental fatigue. Mental fatigue is defined as a psychobiological state caused by prolonged periods of demanding cognitive activity (Boksem et al., 2005). A combination of subjective, behavioral, and physiological manifestations has been used to identify mental fatigue (Van Cutsem et al., 2017), but key outcomes appear to be primarily on a subjective and behavioral level (Russell et al., 2019). On a subjective level feelings of tiredness, lack of energy, decreased motivation and alertness as well as an acute increase of subjective perceptions of effort are linked to mental fatigue (Lorist et al., 2005; Smith et al., 2015; Van Cutsem et al., 2017).

Moreover, impacts of a mental fatigue state can be seen in impairments of executive functions. Executive functions refer to a family of top-down mental processes which are needed when you have to concentrate, pay attention, quickly adapt or change mental strategies, and inhibit response. Executive functions can be divided into three core components including inhibition (e.g., self-control, cognitive inhibition), working memory, and mental flexibility. Regarding this, altered attentional focus (Boksem et al., 2005), slower and less accurate reactions in cognitive tasks (Boksem et al., 2006), and poor use of visual cues for action preparation (Lorist et al., 2000) are the consequence of impairments of executive functions. Thus, prolonged mental exertion negatively influences attention, action monitoring, and cognitive control. It can result in a lack of concentration and alertness as well as in performance decrements (e.g., physical, cognitive, technic-tactical) (Carney et al., 2014; Kellmann and Beckmann, 2018).

Typically, a state of fatigue can be compensated with recovery, which generally contains the reestablishment of invested resources on a physiological and psychological level (Kellmann et al., 2018). To counteract a state of mental fatigue, mentally oriented recovery strategies such as psychological relaxation techniques or resource activation can be used (Kellmann et al., 2018). On this basis, mental recovery aims to obtain baseline levels of mental abilities (e.g., concentration, vigilance, attention) and the restoration of mental energy (Balk et al., 2019). Mental recovery can be achieved through mental resting periods in order to reduce upcoming stress and remove disturbing thoughts. Mental recovery strategies should accelerate the recovery response and can be associated with physiological relaxation effects such as a decrease in muscle-tonus, a decrease in respiratory rate and electro-dermal changes (Dolbier and Rush, 2012; Pelka and Kellmann, 2017). Moreover, mental recovery may lead to an enhancement of mental balance, self-control, and arousal regulation (Loch et al., 2019; Balk and Englert, 2020). Moreover, the idea of mental recovery also underlines the crucial role of self-regulation in the process of mental recovery in relation to the finding and the implementation of the best and adequate recovery for oneself (Beckmann and Kellmann, 2004; Balk and Englert, 2020). Hence, the regulation of thoughts, feelings, and emotions is particularly essential after a mentally stressful situation to recover on a mental level in order to be able to return to the full performance level. Thus, the process of mental recovery coincides with the idea of recovery self-regulation, which can be considered as the process of moving from an actual state (e.g., high mental fatigue, high stress) to a preferred or required future state (e.g., optimal state of recovery) of physical as well as mental activation and readiness by minimizing the discrepancy between both states (Balk and Englert, 2020). Mental recovery can address the different components of mental fatigue (Russell et al., 2019) and is therefore able to accelerate mental recovery as well as to regain performance capacities to enable athletes to train or compete in the next session or competition.

To date, available studies have predominantly investigated the impact of induced mental fatigue on sport-related performance aspects (Van Cutsem et al., 2017; Pageaux and Lepers, 2018), but little is known about the effects and use of mental recovery strategies as a countermeasure of mental fatigue (Rattray et al., 2015). Only a few initial methods have been selected to alleviate mental fatigue such as music (Guo et al., 2015), caffeine ingestion (Van Cutsem et al., 2018), restorative environments (Kaplan, 1995), or the presence of odors (Kato et al., 2012).

Loch et al. (2019) summarize potential mental recovery strategies in a scoping review dealing with the specific topic of mental or psychological recovery strategies in sports (Kudlackova et al., 2013; Keilani et al., 2016; Pelka et al., 2017a,b). Regarding this, strategies such as powernap, systematic breathing, and mental imagery are extracted as potential mental recovery strategies for initial investigation.

Therefore, the purpose of the laboratory study was to investigate acute effects of potential mental recovery strategies (i.e., powernap, systematic breathing, mental imagery vs. control condition) on group-related and individual specific perceptual fatigue, recovery and stress responses as well as on cognitive performance outcomes. We hypothesized (1) that the induction of mental fatigue leads to an increase in the perception of mental fatigue and stress states as well as to a decrease in the recovery states and (2) that the use of mental recovery strategies leads to a counteractive development of the perception of fatigue state, recovery-stress states as well as to an improvement in cognitive performance compared to the control condition.

## Methods

### Participants

Twenty-four female and male undergraduate and graduate students (22.8 ± 3.6 years) participated in the present study, all with a background in recreational or competitive sports. The gender distribution was approximately balanced in the study (14 female, 10 male). The average training volume during a regular week was 6.45 ± 3.22 h. Six participants were mainly involved in team sports (e.g., football, ice hockey, basketball) and 18 participants in individual sports (e.g., track and field, swimming, weightlifting). The range of competition level of participants ranged between regional and international level. Only four of the participants indicated an engagement in mentally-orientated recovery strategies and only one used a recovery strategy in training or competition on a regular basis. All participants were fully informed about the content of the study and provided their written consent before participation. Ethical clearance for the study was obtained by the local ethics committee.

### Study Design and Procedures

**Figure 1:**
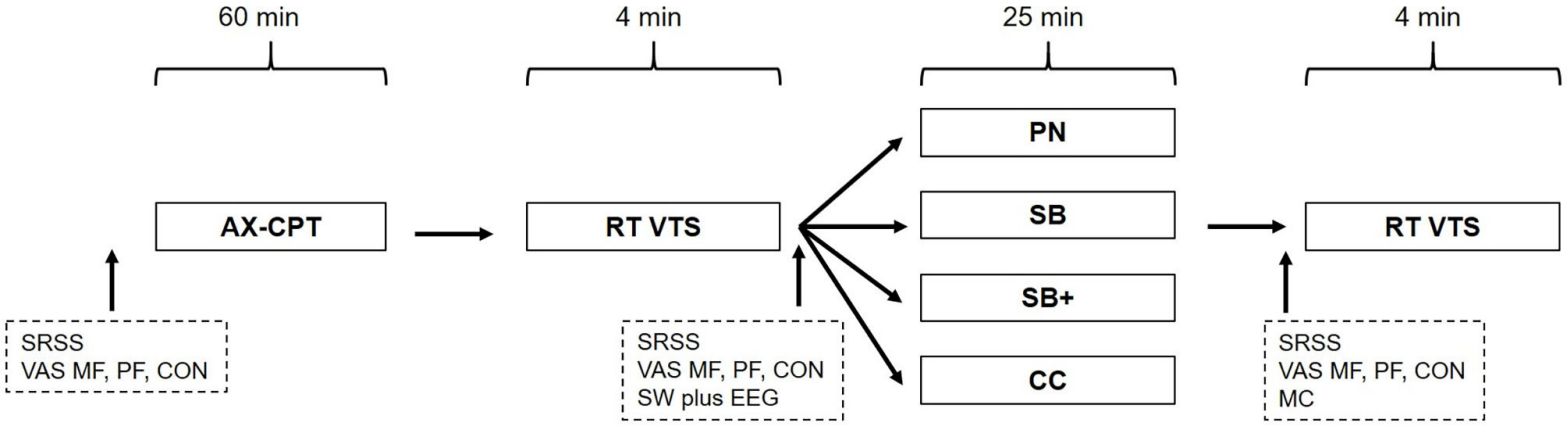
Schematic overview of the study design. AX-CPT, AX-continuous performance test; RT, reaction time test; VTS, Vienna Test System; SRSS, Short Recovery and Stress Scale; VAS, Visual Analog Scale; SW, SOMNOwatch plus EEG; MC, manipulation check; PN, powernap; SB, systematic breathing; SB+, systematic breathing plus mental imagery; CC, control condition.

A randomized cross-over design with repeated measurements was used in which the order of intervention groups and control conditions was counterbalanced. The laboratory-based study comprised four consecutive intervention sessions, in which participants visited the test rooms once a week on the same working day and in the same time slot (Figure 1). In preparation for the study, the participants were able to choose between one of three different time slots (12.00–2.00 p.m.; 1.15–3.15 p.m.; 2.45–4.45 p.m.) which were linked to the single participants, respectively. The random assignment of the participants to respective intervention orders was based on algorithmic calculation.

At the beginning of the first intervention session, demographic data was collected and participants were asked to complete a selection of questionnaires including the Short Recovery and Stress Scale (SRSS) (Kellmann et al., 2016; Kellmann and Kölling, 2019) and different Visual Analog Scales (VAS) to assess subjective ratings of *Mental Fatigue* (MF), *Physical Fatigue* (PF), and *Concentration* (CON) (Lee et al., 1991). In addition, experiences with mentally orientated recovery strategies of the participants were captured.

Next, participants completed a 60-min AX-continuous performance test to induce mental fatigue. After this, participants were run through a 4-min single reaction time test on the Vienna Test System (VTS). The SRSS and VAS were provided for the second time, before participants were relocated to the relaxation room, which was darkened, contained no windows, and was fixed to constant climate which did not change throughout the different interventions. For the following 25 min, participants underwent one of three mental recovery interventions [powernap (PN), systematic breathing (SB), systematic breathing plus mental imagery (SB+), and the control condition (CC)]. During each mental recovery session, portable polysomnography (SOMNOwatch plus EEG, Somnomedics, Randersacker, Germany) was used to measure specific sleep-related parameters [e.g., Total Sleep Time (TST); Voinescu et al., 2014; Hof zum Berge et al., 2019]. Following the recovery intervention, the SRSS and VAS were completed for the third time. Finally, participants repeated the reaction time test as previously conducted. A manipulation check was implemented to provide feedback on efficacy, appreciation, and feasibility of the performed mental recovery strategies. To ensure engagement and vigilance during the mental fatiguing task, an award was guaranteed for the three best overall performances during the AX-CPT and reaction time test.

### Induction of Mental Fatigue

In laboratory settings, mental fatigue is commonly induced by prolonged engagement in demanding cognitive tasks over a period of 30 up to 90 min (Van Cutsem et al., 2017; Pageaux and Lepers, 2018). To generate a state of mental fatigue, cognitive tasks should be used for a minimum duration of 30 min (Pageaux and Lepers, 2018). Therefore, mental fatigue was induced by completion of the 60-min version of the AX-continuous performance test (AX-CPT) (Guo et al., 2015). This demanding cognitive task requires vigilance, working memory, and response inhibition, and it has been used successfully to induce a state of mental fatigue in previous studies (Marcora et al., 2009; Pageaux et al., 2013; Smith et al., 2015). The AX-CPT is configurated so that sequences of letters are visually presented one at a time in a continuous manner on a computer screen. Participants were instructed to press one of two keys on target trials (defined as a cue-probe sequence in which the letter *A* appeared as a cue and the letter *X* appeared as a probe) or the other key otherwise. The other letters of the alphabet served as invalid cues or non-target probes. AX-CPT performance was scored automatically on the basis of reaction time and accuracy of responses. As an immediate performance feedback indicator, any missed or incorrect responses resulted in a beep sound from the computer speakers acted as a probe to increase speed and accuracy.

### Psychological Measures

Prior to the first intervention session, basic demographic data (e.g., age, hours spent doing sports, type of sport) were gathered. The SRSS is composed of eight items and is categorized into four recovery-related [*Physical Performance Capability* (PPC), *Mental Performance Capability* (MPC), *Emotional Balance* (EB), and *Overall Recovery* (OR)] and four stress-related [*Muscular Stress* (MS), *Lack of Activation* (LA), *Negative Emotional State* (NES), and *Overall Stress* (OS)] items. Answers were provided on a 7-point Likert Scale (0–6) ranging between “*does not apply at all”* and “*fully applies.”* The eight scores of the SRSS have shown acceptable internal consistencies, ranging from α = 0.70 to α = 0.76 (Kellmann et al., 2016; Kellmann and Kölling, 2019).

In addition, MF, PF, and CON were rated using 100-mm Visual Analog Scales (VAS) (Smith et al., 2015; Vrijkotte et al., 2018). These instruments were used to measure the perceptions of mental fatigue and mental recovery of the participants over the period of the intervention session. A VAS is a common instrument to assess the state of mental fatigue and is therefore frequently applied in experimental studies. The VAS has been reported as a valid and reliable instrument to measure mental fatigue (Lee et al., 1991). Participants were asked to rate the extent to which they felt mentally and physically fatigued and to which extent they felt they could focus on three different scales (ranging from “*not at all”* to “*completely”*). No other markings were displayed on the scales. Participants were asked to mark the point that represented their perception of the current state.

A self-designed manipulation check was administered after each intervention session to gain insight into participants' evaluations of the interventions. The manipulation check addressed how participants experienced the mental recovery period on three different items. Both the efficacy (*As how effective did you experience the recovery method?*) and the appreciation (*How did you like the recovery method?)* of the mental recovery interventions were evaluated by the participants on 7-point Likert Scales (1–7), ranging from “*not at all”* to “*fully applies.”* The third question measured the feasibility of the current intervention method (*I was able to apply the mental recovery method correctly)* on a 7-point Likert Scale (1–7), ranging from *not at all implemented* to *perfectly implemented*.

### Cognitive Performance

Cognitive performance was assessed using a simple reaction time test (RT, version S1) of the Vienna Test System (VTS, Schuhfried, Moedling, Austria). The test period takes 4 min in total, during which the participant has to react as quickly as possible to an optical signal (yellow dot). A key is pressed and released at maximum speed possible when seeing a simple stimulus (yellow light). The participants run one test round to understand the task before actually taking the reaction test. During the test period, 28 stimuli are presented. Mean reaction time (MRT) and mean motor time (MMT) are measured to assess cognitive performance on the task. High reliability values were found for RT/S1 with Cronbach's alpha of at least 0.83 for mean reaction time and 0.84 for mean motor time.

### Mental Recovery Interventions

The selection of interventions was mainly based on the procedures and findings of Pelka et al. (2017b). During the recovery period of 25 min PN, SB, and SB+ were introduced. The PN intervention consisted of an introduction period and a 20-min nap. All participants were instructed to nap on a bed in a comfortable lying position. The SB intervention was composed of an introduction period, a main part, and a retrieval period. A breathing rhythm in which the exhalation phase was required to be twice as long as the inhalation period (leading to a 3/6 or 4/8s rhythms), was applied. As a third strategy, participants performed a systematic breathing intervention that was combined with mental imagery (combination of systematic breathing and self-selected relaxation picture). For the mental imagery intervention the introduction period and a shortened main part of the systematic breathing intervention were adopted and linked to an imaginative period in which the participants visualized a self-selected relaxing picture (e.g., relaxing atmosphere at the beach; Martin et al., 1999). In the SB as well as the SB+ intervention, participants were guided through a pre-recorded audio instructions. In the control condition participants had the possibility to read a collection of comics. The choice of comics as CC was made based on similar usage in previous research (Pelka et al., 2017b). Additional interventions instructions were verbally given by an educated researcher.

### Data Analyses

A power analysis was conducted using G^*^Power (parameters: repeated measures ANOVA, within-between interaction, *f* = 0.25, *p* = 0.05, power = 0.80, number of groups: 4, number of measurement points: 3) yielding a sample size of 32 participants. For the present study, 32 participants could be recruited, but due to the exclusion of eight participants only 24 participants could be included in the analyses.

Data were analyzed using R Studio software (R Foundation, V.1.1.423) and Microsoft Excel 2019. Data are presented as mean ± SD. Assumption of normality was confirmed by means of Shapiro-Wilk-Test before the conduction of any parametric test. Repeated measures analysis of variance (RM-ANOVA) with factors recovery interventions and time were used to verify the induction of mental fatigue and to determine differences in all analyzed parameters (i.e., fatigue states, recovery-stress states, cognitive performance) between recovery interventions as well as measurement points. When the sphericity assumption was violated, the Greenhouse-Geisser correction was employed. Cohen's effect sizes (*d*) were calculated and interpreted using thresholds of 0.2, 0.5, 0.8 for small, moderate and large effects, respectively (Cohen, 1992).

To present individual specific results and to evaluate practical relevance, individual standardized differences [i.e., individual standardized changes between measurement points (pre-MR to post-MR) and standardized differences in changes between intervention groups and control condition] were calculated as effect sizes (ES) using the individual pooled pretest standard deviation (SD). In subjective ratings of VAS the smallest worthwhile change (SWC) was set as a 10 mm change on the VAS. In SRSS, the SWC was defined as a minimum change of 1.0 per Item. The threshold values of 0.00–0.19, 0.20–0.59, 0.60–1.19, 1.20–1.99, and ≥2.00 were considered trivial, small, moderate, large, and very large, respectively.

To monitor whether participants followed the instructions of the respective recovery intervention (i.e., participants have slept/participant have not slept), a portable polysomnography system (SOMNOwatch plus EEG) was applied, measuring frontal brain derivation, as well as eye-movement via electrooculography (EOG) and muscle tension via electromyography (EMG) (Voinescu et al., 2014; Hof zum Berge et al., 2019). Sleep stages were manually scored using 30-s epochs according to the AASM guidelines (Berry et al., 2012) with adaptations to the single-EEG-device based on the criteria suggested by Lucey et al. (2016). As exclusion criteria, we set a total sleep time of 2 min or greater during the rest period in all four intervention groups. Analysis of recorded Total Sleep Time (TST) revealed that twelve participants of the PN group, three participants of the SB group, four participants of the SB+ group and none of the CC group have to be excluded. For all further analysis the adjusted group sizes were used.

## Results

To verify whether the AX-continuous performance test effectively induces a state of mental fatigue, RM-ANOVA revealed significant time effects for the VAS MF from pre-AX-CPT to pre-MR [*F*_(1, 8)_ = 40.05, *p* < 0.001, $\eta_{\text{p}}^{2}$ = 0.83], but no differences between groups, or interactions between groups and measurement points could be found. In contrast, for the values of VAS PF, RM-ANOVA showed no significant change over time from pre-AX-CPT to pre-MR. Considering the ES statistics, the AX-CPT test induced large to very large increases between pre- and post-measurement points in the perception of mental fatigue (PN: *d* = 2.21, SB: *d* = 1.15, SB+: *d* = 1.03, CC: *d* = 1.64).

**Figure 2:**
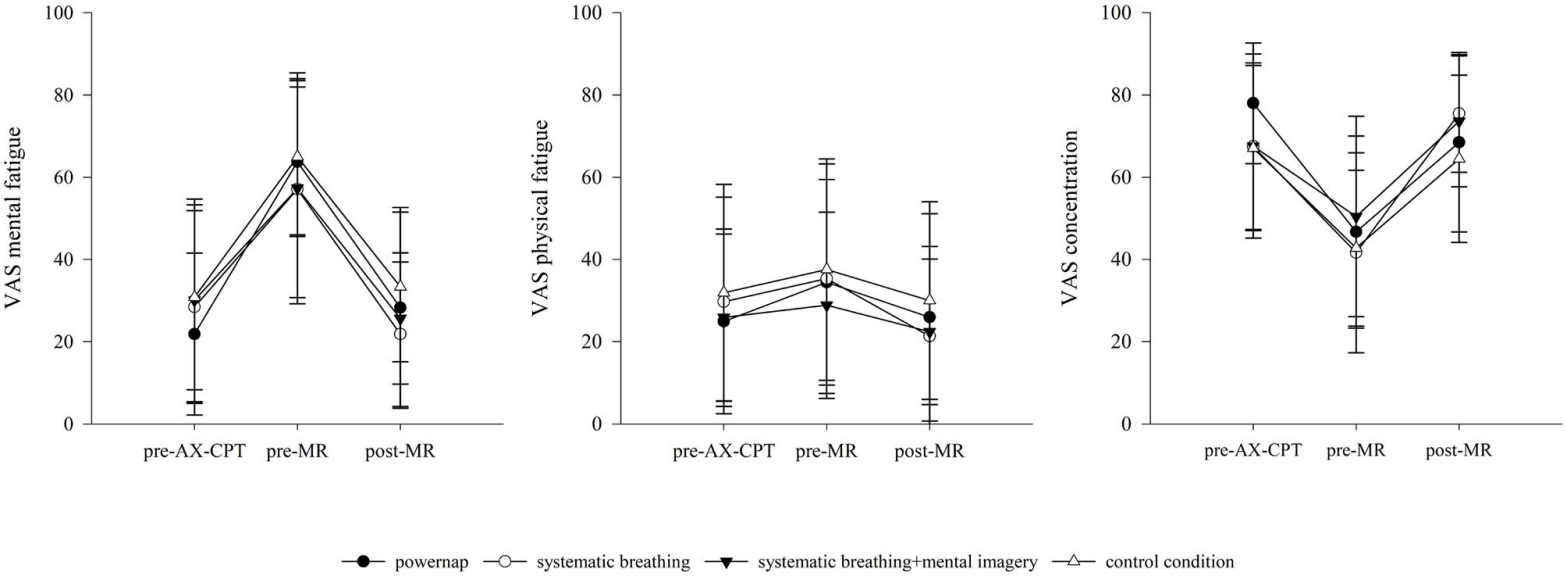
Descriptive changes of VAS (MF, PF, CON) over the course of an experimental day. AX-CPT, AX-continuous performance test; MR, Mental recovery. Results presented as mean ± SD.

For the general and mental specific items of the SRSS, RM-ANOVA showed significant time effects from pre-MR to post-MR for OS scores [*F*_(1, 8)_ = 28.75, *p* < 0.001, $\eta_{\text{p}}^{2}$ = 0.78], OR scores [*F*_(1, 8)_ = 36.66, *p* < 0.001, $\eta_{\text{p}}^{2}$ = 0.82], MPC scores [*F*_(1, 8)_ = 32.50, *p* < 0.001, $\eta_{\text{p}}^{2}$ = 0.80], and LA scores [*F*_(1, 8)_ = 42.09, *p* < 0.001, $\eta_{\text{p}}^{2}$ = 0.84], however no differences between groups nor interactions between groups and measurement points appeared (Figure 2).

**Figure 3:**
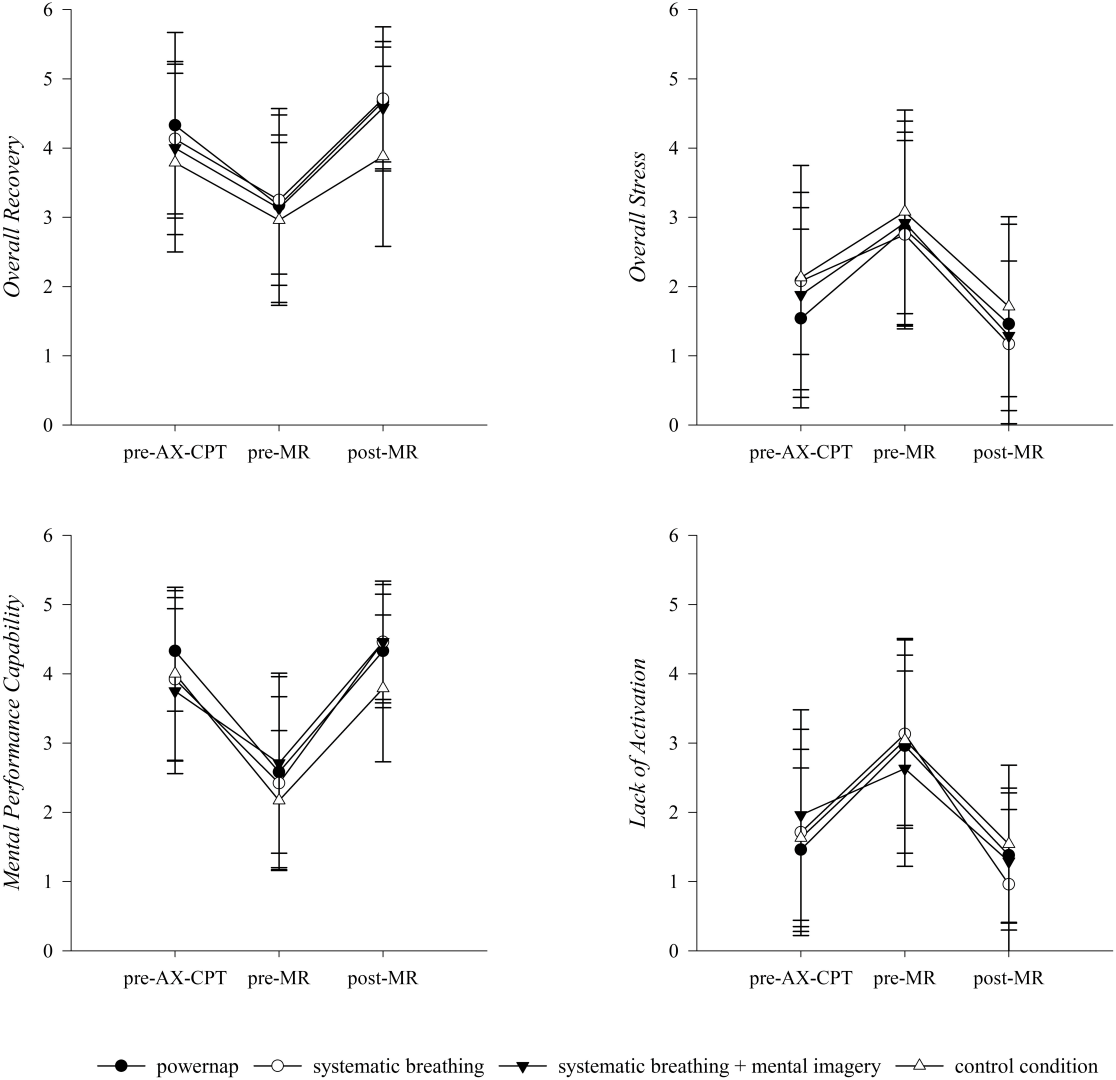
Descriptive development of general and mental specific items of the SRSS over the course of an experimental day. AX-CPT, AX-continuous performance test; MR, Mental recovery. Results presented as mean ± SD.

Regarding the physical and emotional specific items of the SRSS, values of PPC [F_(1, 8)_ = 31.88, *p* = < 0.001, $\eta_{\text{p}}^{2}$ = 0.80] and EB [*F*_(1, 8)_ = 8.45, *p* < 0.05, $\eta_{\text{p}}^{2}$ = 0.51] changed significantly over time from pre-MR to post-MR. In addition, a significant group x time interaction was found for the values of the PPC [*F*_(3, 24)_ = 3.21, *p* < 0.05, $\eta_{\text{p}}^{2}$ = 0.29]. For the items of MS and NES no significant results could be revealed (Figure 3).

**Figure 4:**
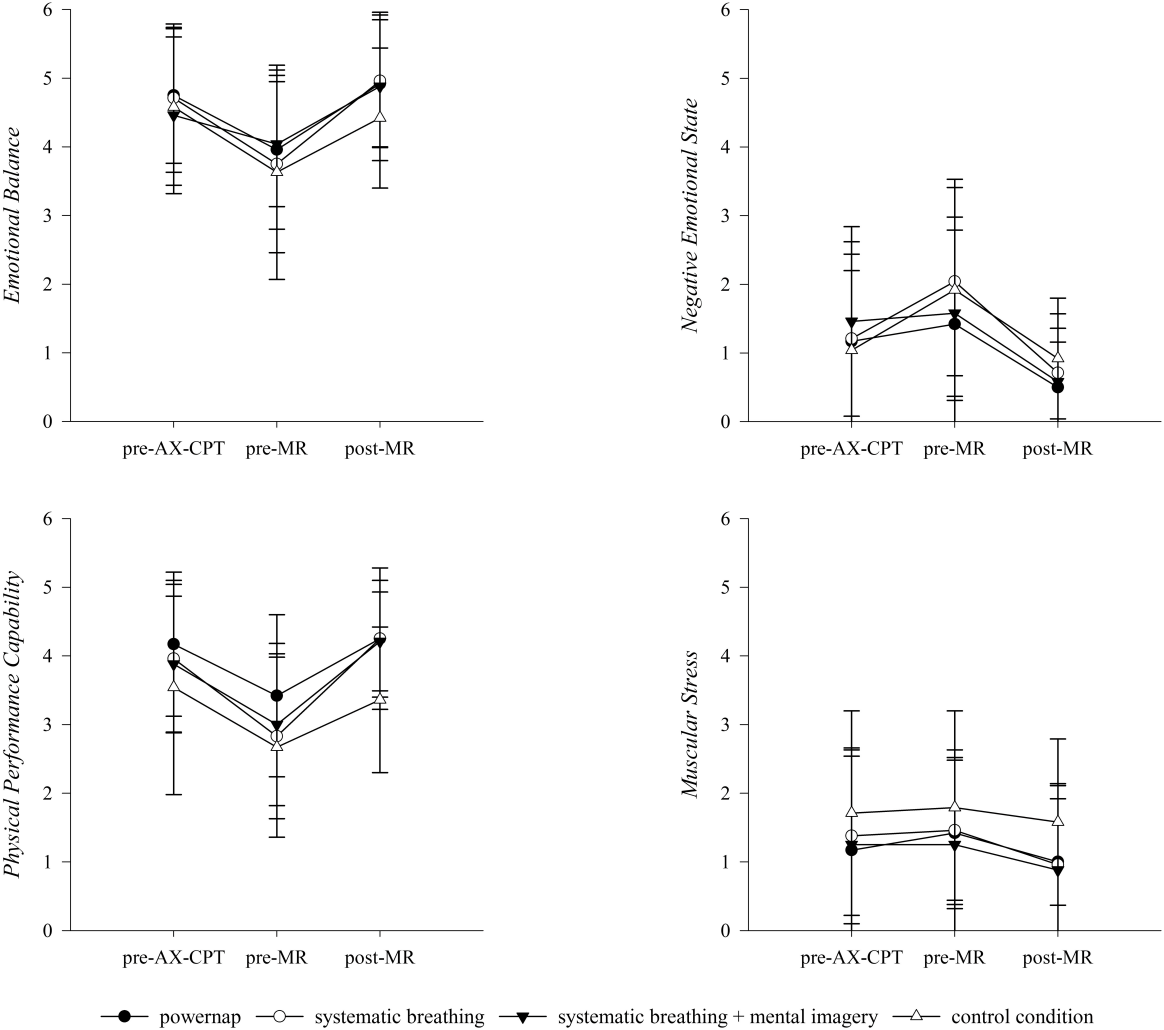
Descriptive development of the emotional and physical specific items of the SRSS over the course of an experimental day. AX-CPT, AX-continuous performance test; MR, Mental recovery. Results presented as mean ± SD.

For the subjective ratings of VAS MF and VAS CON significant differences over time from pre-MR to post-MR were found [MF: *F*_(1, 8)_ = 29.42, *p* < 0.001, $\eta_{\text{p}}^{2}$ = 0.79; CON: *F*_(1, 8)_ = 34.71, *p* < 0.001, $\eta_{\text{p}}^{2}$ = 0.81]. No differences between groups and no interaction between groups and measurement points were observed. The results of the VAS PF revealed a significant group x time interaction from pre-MR to post-MR [PF: *F*_(3, 24)_ = 3.04, *p* < 0.05, $\eta_{\text{p}}^{2}$ = 0.28]. Changes of VAS MF, PF, and CON over the course of an experimental day are illustrated in Figure 4.

The results of the manipulation check of recovery interventions did not indicate significant differences related to self-rated efficacy, appreciation, and feasibility of the three mental recovery interventions and CC. Relating to the efficacy of the implemented interventions, descriptive data obtained higher scores for mental recovery interventions (PN: 6.09 ± 0.83; SB: 5.75 ± 1.25; SB+: 6.00 ± 0.92) compared to the CC (4.48 ± 1.68). Moreover, results showed that the feasibility of the mental recovery interventions (PN: 4.91 ± 0.70; SB: 4.35 ± 1.09; SB+: 4.60 ± 0.82) were assessed as lower compared to the CC (5.26 ± 0.75).

A main effect of time was found for the comparison between pre-MR and post-MR mean reaction time [MRT: *F*_(1, 8)_ = 11.54, *p* < 0.05, $\eta_{\text{p}}^{2}$ = 0.62] and mean motor time [MMT: *F*_(1, 8)_ = 19.74, *p* < 0.01, $\eta_{\text{p}}^{2}$ = 0.74]. However, no differences between groups and interaction between groups and measurement points were observed. For all of the three mental recovery interventions the analyses yielded that participants significantly improved their mean reaction time from pre- to post-MR with small effect sizes [PN: ES = −0.32,SB: ES = −0.19; SB+: ES = −0.29, CC: ES = −0.25]. Again, participants significantly improved their mean motor time from pre- to post-MR in all of the three mental recovery interventions [PN: ES = −0.35; SB: ES = −0.28; SB+: ES = −0.34; CC: ES = −0.37; Table 1).

**Table 1:** Descirptive changes in mean reaction time and mean motor time pre- and post-mental recovery interventions.

**Interventions**	**Pre-MR MRT (ms)**	**Post-MR MRT (ms)**	**Pre-MR MMT (ms)**	**Post-MR MMT (ms)**
PN	267.83 (±44.85)	253.92 (±41.17)	135.00 (±31.74)	123.50 (±34.38)
SB	269.48 (±46.56)	260.86 (±42.65)	133.33 (±51.30)	120.90 (±35.61)
SB+	262.40 (±32.40)	252.75 (±35.04)	125.55 (±27.99)	115.85 (±29.60)
CC	264.67 (±43.23)	254.96 (±35.64)	129.67 (±40.87)	116.08 (±31.38)

*PN, powernap; SB, systematic breathing; SB+, systematic breathing + mental imagery; CC, control condition; MR, Mental recovery; MRT, Mean reaction time; MMT, Mean motor time*.

*Results presented as mean ± SD*.

**Figure 5:**
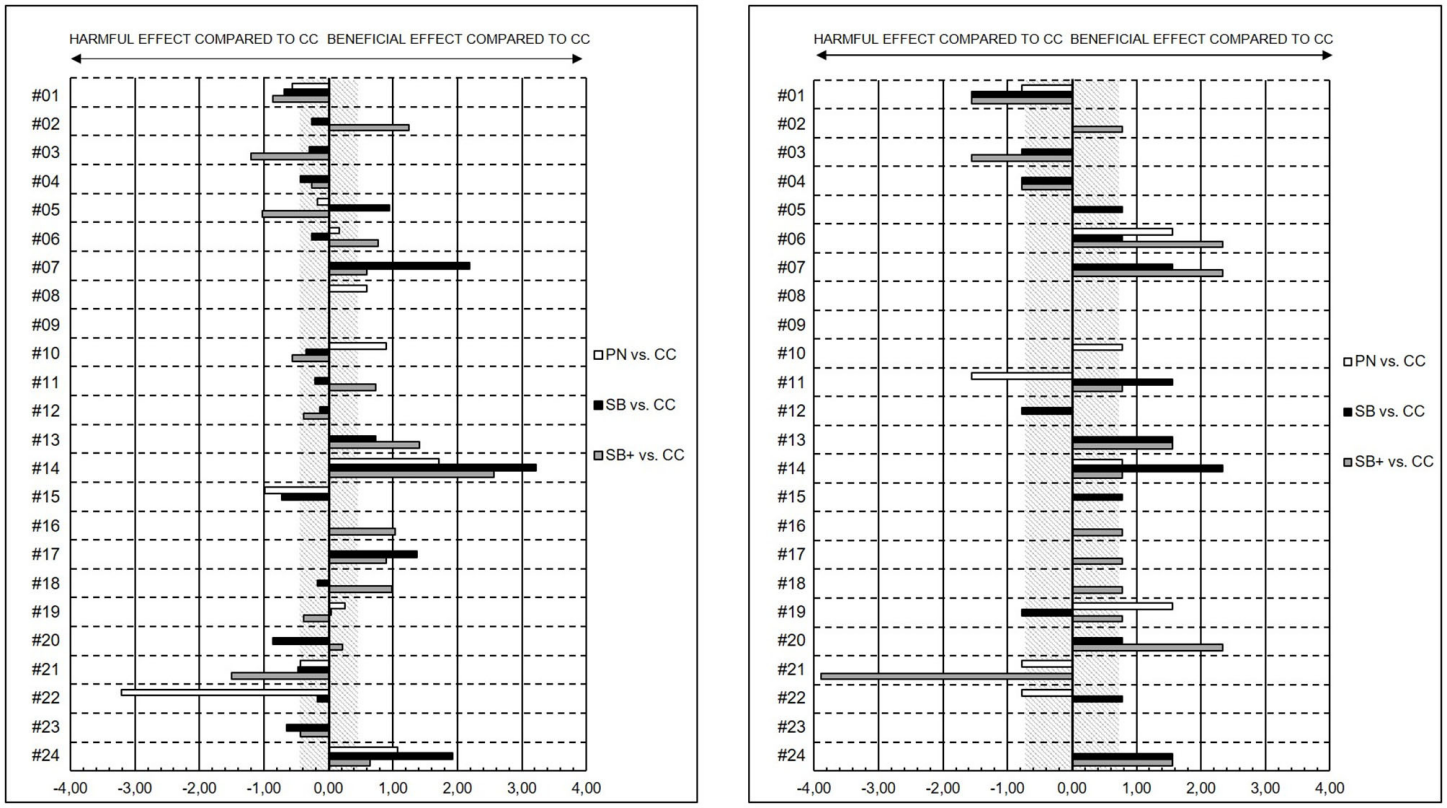
Individual standardized differences in the pre-MR to post-MR changes presented as effect sizes (ES) between powernap (PN), systematic breathing (SB), systematic breathing plus mental imagery (SB+), and control condition in the VAS MF **(left)** and in MPC **(right)**. The gray stripped areas represent the smallest worthwhile change (SWC) in the VAS MF as well as in the MPC.

**Figure 6:**
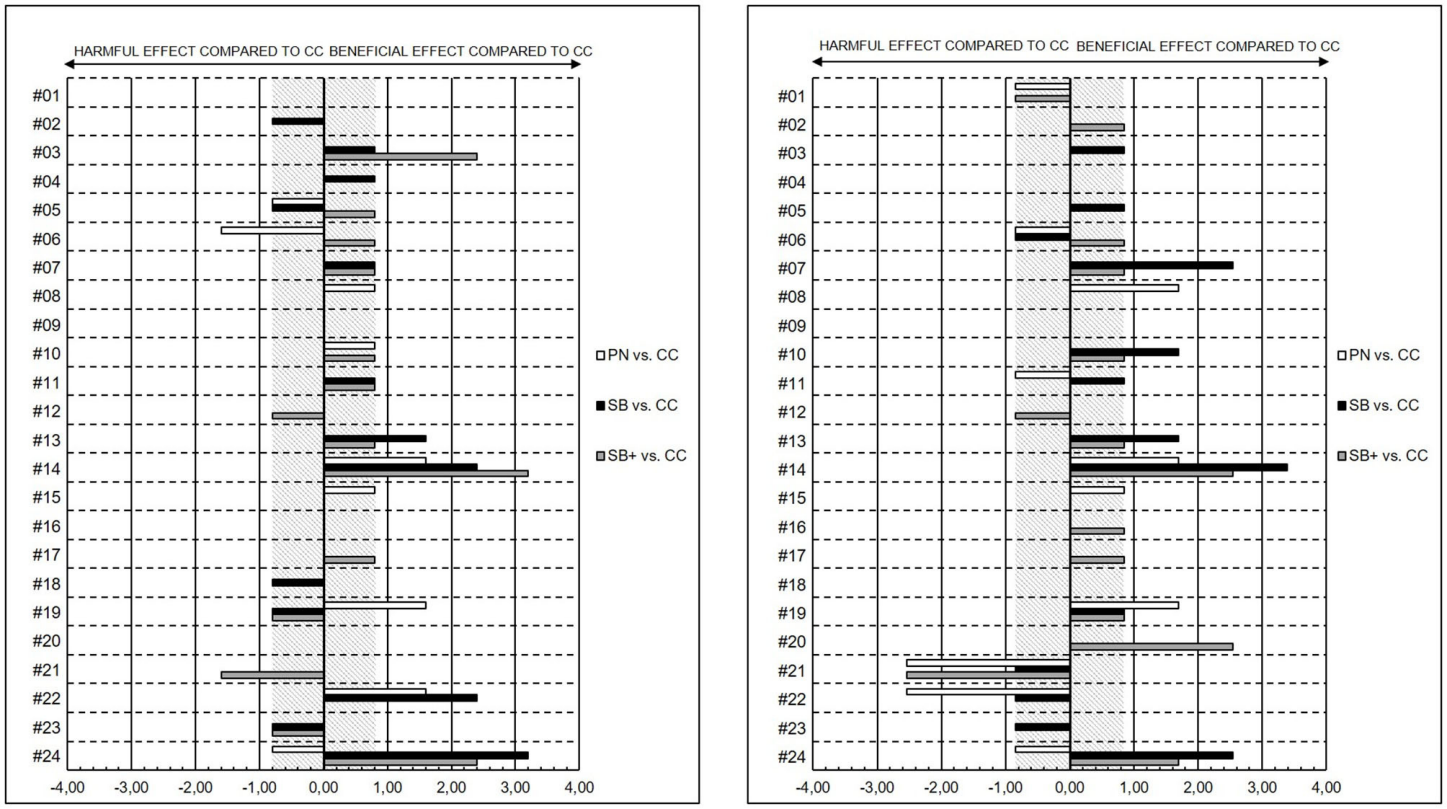
Individual standardized differences in the pre-MR to post-MR changes presented as effect sizes (ES) between powernap (PN), systematic breathing (SB), systematic breathing plus mental imagery (SB+) and control condition in the OR **(left)** and in the PPC **(right)**. The gray stripped areas respresent the smallest worthwhile change (SWC) in the OR as well as in the PPC.

With the focus on individual responses of PN, SB and SB+ compared to CC on fatigue states, recovery-stress states, and cognitive performance, results indicated mainly disparate magnitudes of changes, but revealed substantial individual specific differences in the changes in VAS MF, MPC, OR, and PPC. Individual standardized differences in the pre-MR to post-MR changes of selected items of SRSS and VAS are shown in Figures 5, 6.

Regarding some individual results, for subject #14 all mental recovery strategies showed beneficial effects on VAS MF (PN vs. CC: ES = 1.71; SB vs. CC: ES = 3.21; SB+ vs. CC = 2.57), MPC (PN vs. CC: ES = 0.78; SB vs. CC: ES = 2.33; SB+ vs. CC: ES = 0.78), OR (PN vs. CC: ES = 1.60; SB vs. CC: ES = 2.40; SB+ vs. CC: ES = 3.20), and PPC (PN vs. CC: ES = 1.69; SB vs. CC: ES = 3.39; SB+ vs. CC: ES = 2.54) compared to CC. In comparison to SB and SB+, the magnitude of change of subject #19 revealed beneficial effects for PN on MPC (PN vs. CC: ES = 1.56), OR (PN vs. CC: ES = 1.60) and PPC (PN vs. CC: ES = 1.69). In contrast, for subject #21, all mental recovery strategies showed no to harmful effects on VAS MF (PN vs. CC: ES = −0.43; SB vs. CC: ES = −0.47; SB+ vs. CC = −1.50), MPC (PN vs. CC: ES = −0.78; SB vs. CC: ES = 0.00; SB+ vs. CC: ES = −3.89), OR (PN vs. CC: ES = 0.00; SB vs. CC: ES = 0.00; SB+ vs. CC: ES = −1.60) and PPC (PN vs. CC: ES = −2.54; SB vs. CC: ES = −0.85; SB+ vs. CC: ES = −2.54) compared to CC.

## Discussion

Short recovery periods are mainly targeting the psychophysiological recovery (Pelka et al., 2017a) emphasizing that a multifaceted recovery process on both psychological and physiological level is essential. Multifaceted demands can result in mental fatigue which consequently implies that recovery strategies are needed to reduce mental fatigue on a psychological level (Pelka et al., 2017b; Kellmann and Beckmann, 2018).

However, scientific evidence of the necessity for mental recovery is lacking, this laboratory-based study, therefore, aimed at assessing acute effects of the induction of mental fatigue. It further investigated acute effects of mental recovery strategies on perceptual fatigue and recovery-stress states as well as on cognitive performance measures as a countermeasure to a state of mental fatigue. Subjective and objective data (i.e., SRSS, VAS, manipulation check, mean reaction time, mean motor time) were examined in order to detect differences over time, between interventions and in individual responses.

As the AX-CPT has previously been identified as mentally fatiguing (Marcora et al., 2009; MacMahon et al., 2014; Smith et al., 2015) we used the 60-min version of the AX-CPT to induce mental fatigue. From pre- to post-AX-CPT, all recovery interventions as well as the control condition exhibited subjective signs of mental fatigue. Results of the VAS MF showed significantly higher values pre-MR compared to the initial state (pre-AX-CPT). Moreover, the mean pre-MR ratings of VAS MF (60.78 ± 22.92) found in the present study are similar to those reported in other studies using the AX-CPT (Pageaux et al., 2013; Smith et al., 2015). By contrast, no significant difference in ratings of VAS PF were revealed from pre-AX-CPT to pre-MR and values were considerably lower than values of VAS MF illustrating that participants may differentiate between the separate facets of fatigue on a primarily mental level compared to a physical level. In addition, results revealed a significant increase in stress-related items of the SRSS (OS, LA, NES*)* and a significant decline in recovery-related items of SRSS (OR, MPC, EB, PPC) for all recovery groups as well as the control condition. As expected, these results indicate that the AX-CPT successfully induced a state of mental fatigue as demonstrated by an increase in perception of mental fatigue and stress states as well as a decrease in recovery states (Guo et al., 2015; Smith et al., 2015).

Against our expectations, it has to be underlined that the analysis yielded no significant differences between recovery interventions and the control condition. One possible reason for this might be that participants even perceived the control condition as recovering on a mental and emotional level, which leads to a similar perception of fatigue and recovery-stress states. This could be due to the fact that the control condition comprised moderate mental demands with giving no systematic guidance compared to the implemented mental recovery strategies. Focusing on acute effects of mental recovery strategies, results showed significant time effects for SRSS items, VAS and cognitive performance outcomes from pre- MR to post-MR. As expected, changes revealed that the implemented mental recovery strategies had a positive impact on participants' perceptions of their fatigue and recovery-stress states which were basically reflected by an increase of OR and a decline of OS and MF scores. Since rest periods primarily aim at a re-establishment of pre-performance states as well as personal resources (Robson-Ansley et al., 2009; Pelka and Kellmann, 2017) and mental recovery includes both mental and emotional aspects of recovery (Balk et al., 2018), the results illustrate that a rest period with applying any of the mental recovery strategies subject to this study leads to improvements of MPC, EB and the perception of CON as well as to reductions of LA values and the perception of MF in response to a mentally fatiguing task. Moreover, results show an increase in PPC from pre-MR to post-MR, contrarily perceptions of MS and PF remained mainly linear. These findings are in line with the general understanding of the recovery process, assuming that the application of recovery strategies activate both physical as well as mental components (Pelka and Kellmann, 2017; Kellmann et al., 2018). However, current results indicate that the application of a “mental break” during which mental as well as emotional components have been taxed, can return to their baseline levels (Balk et al., 2018) and may allow participants to switch off from previous mental demands. Because of the fact that manifestations of mental fatigue are primarily associated with subjective and behavioral markers such as feelings of tiredness, lack of energy, and perception of increased effort (Van Cutsem et al., 2017; Kölling et al., 2019), results suggest that a “mental break” can buffer mental fatigue outcomes by increasing mental and emotional recovery states (i.e., OR, MPC, CON, EB) with corresponding feelings of being physically and mentally recovered, concentrated, receptive, alert, and balanced (Eccles and Kazmier, 2019; Kellmann and Kölling, 2019).

Since recovery is a highly individual process and recovery strategies have to match an individual's specific needs (Pelka et al., 2017b; Heidari et al., 2018), missing intervention effects of the laboratory study can also be explained. Optimal short-term recovery can only be achieved when recovery activities are consciously planned according to situational and environmental needs (Pelka and Kellmann, 2017). With regard to the distinction between the idea of rest and mental recovery, rest (e.g., reduction of cognitive demands, physical rest, inactivity) might be important in promoting recovery, but the systematic use of mental recovery modalities (e.g., systematic breathing, mental imagery, detachment) appears to be more beneficial to recover on a mental and emotional level (e.g., regulation of thoughts and feelings, regulation of psychophysiological activation). Particularly, the reduction of mental stress and the disengagement of cognitively stressful situations (e.g., process of mental detachment) during the period of mental recovery appears to be beneficial to activate and promote the process of mental recovery (e.g., regulation of psychophysiological activation, prevention of sustained rumination, enabling positive, and goal-orientated emotions/thoughts; Balk et al., 2018, 2019; Eccles and Kazmier, 2019). Thus, focusing on rest periods, consciously used (pro-active) mental recovery strategies appear to be beneficial to reestablish essential resources and to counteract mainly subjective facets of mental fatigue in order to maintain performance readiness on a physical, mental, and emotional level (Smith et al., 2018).

Regarding the cognitive performance, several previous studies have shown that short naps in particular can improve vigilance and alertness as well as features of mental and physical performance (e.g., cognitive and motor performance aspects; Waterhouse et al., 2007; Ficca et al., 2010; Debarnot et al., 2011). Our findings confirm these results as they revealed improvements in cognitive performance measures with a reaction time test on the VTS. Participants were able to increase mean reaction time and mean motor time from pre-MR to post-MR. However, training effects from pre- to post-measurement points have to be taken into account. In this context, with respect to the effects of mental fatigue on subjective as well as performance outcomes, the impact of motivation and self-efficacy has to be mentioned. To include the potential role of motivation in the study design, we determined an award to ensure engagement and vigilance during the AX-CPT. Although the role of motivation in the development of mental fatigue is still unclear, it should be considered that the engagement in a cognitively demanding, self-initiated and predominantly boring task (AX-CPT) over 60 min may affect self-control abilities and could result in a loss of motivation as well as in a lower level of self-efficacy depending on the individual perception of performance (Englert, 2016). Therefore, changes regarding the participants' motivation status could also be a potential explanation for performance decrements (e.g., impact on the performance in the reaction time test following the AX-CPT). In order to get more information on this aspect, the integration of measures of motivation and self-efficacy can be an interesting and useful addition in further studies.

Descriptive results of the manipulation check underline that participants perceived the mental recovery interventions as recovering and supportive to regain resources primarily on a mental and emotional level. Nonetheless, no significant differences between the interventions could be found. Relating to the feasibility of the different interventions, participants could realize the control condition much better than the other three interventions. Taking into account the fact that participants only have minimal experience with mental-orientated recovery strategies, it underlines that the feasibility of recovery strategies is mediated by both previous experience with recovery interventions and by how comfortable one feels with the specific recovery strategy (Pelka and Kellmann, 2017). This is supported by the results of PSG-analyses showing that less than half of the participants were able to nap in a rest period of 20 min. Difficulties in feasibility of used mental recovery strategies might be explained by the fact that some participants were not able to fall asleep due to new surroundings and lack of practice. Conversely, some participants, were able to fall asleep due to an individual high sleep pressure. Although there is a lack of clarity with regard to specific durations of a powernap (e.g., 10-min vs. 20-min naps overall), taking the ecological validity (e.g., feasibility in fixed between-competition rest periods) into account, it has to be discussed whether brief naps with a specific duration of sleep are necessary or whether a period of rest in comfortable position and quiet conditions is sufficient.

Finally, some limitations of the study design must be considered. First, the high drop-out rate of participants due to missing test days and missing or incorrect test values has to be considered regarding the classification of the results of the present study. A *post hoc* power analysis using the same parameters and 24 participants was calculated resulting in a power of 0.69, which decreases the probability of actually detecting if the effect is true; there is a higher chance of false positive effects. With regard to the individual character of mental recovery and because of the structured and especially individualized manner of mental recovery strategies, these strategies need to be tested by each individual in order to decide which are appropriate. In relation to this assumption, the presented results of the present pilot study, however, underline the additional benefits in this specific setting. Second, the screening of TST with SOMNOwatch plus EEG is reasonable, but led to the exclusion of several participants on the intervention groups. Third, in the participants' pre-experiment states, no initial cognitive performance test (i.e., baseline measurement point) was administered, meaning changes of MRT and MMT from baseline to post-AX-CPT and post-MR measurement points could not be included in the analysis. Fourth, no additional physiological and hormonal parameters such as heart rate measures (HR, HRV), respiratory measures or cortisol levels were obtained from the participants during interventions. Additional data are required to allow for more detailed information about psychophysiological responses of mental recovery strategies apart from subjective outcomes to draw more accurate conclusions. Fifth, as already mentioned, the choice of comics as an appropriate control condition needs to be discussed for the use in further studies investigating effects of mental recovery strategies. Moreover, it has to be underlined that no examination was applied during the control condition to check whether the participants read some of the comics or only used the recovery break of 25 min to rest.

## Conclusion and Future Directions

Keeping in mind that recovery is a highly individual process, the results of the laboratory-based study revealed significant time effects for SRSS items, VAS and cognitive performance outcomes but no significant differences between mental recovery interventions and control conditions could be found. However, following the induction of mental fatigue, the use of mental recovery strategies leads to a counteractive development of the perception of fatigue and recovery-stress states. Following this first advance, it could be derived that the application of a rest break with 20 min of mental recovery may enhance recovery on a mainly mental and emotional level.

Regarding this, further studies are necessary to test complementary effects of these strategies in an applied sport setting with athletes of different levels and sports and to gain novel insights in the field of mental recovery. Sports with multiple competition bouts throughout a day would be of particular interest for future research. Rest periods (e.g., 20 min) between separate competitions appear to be beneficial for athletes to cope with the mental demands of competitions and to become more resilient to the negative facets and impairments of mental fatigue (Van Cutsem et al., 2017). Therefore, further studies should evaluate whether rest periods with shorter or longer recovery time will lead to greater or smaller effects compared to a 20-min rest period. Hence, in future studies, additional relevant perceptual, physiological, and hormonal parameters as well as sport-specific performance parameters could be assessed to get a more holistic view of the effects of mental recovery. Because of the fact that recovery depends on previous activities and on specific performance characteristics, it is also conceivable to integrate additional recovery strategies with predominant active parts (e.g., adapted cool-down, short, and moderate walks, adapted exercise routines) in future studies.

## Data Availability Statement

The raw data supporting the conclusions of this article will be made available by the authors, without undue reservation.

## Ethics Statement

The studies involving human participants were reviewed and approved by the Ethics Committee of the Faculty of Sport Science, Ruhr University Bochum. The participants provided their written informed consent to participate in this study.

## Author Contributions

FL prepared the original manuscript, figures and tables. FL and AH analyzed the data and interpreted the results. AH and MK assisted with writing and editing the manuscript, figures and tables. AH, TM, MP, AF, and MK conceived and designed the experiment. All authors contributed to the article and approved the submitted version.

## Conflict of Interest

The authors declare that the research was conducted in the absence of any commercial or financial relationships that could be construed as a potential conflict of interest.
